# Factores determinantes sociales de la salud: la investigación que necesitamos.

**DOI:** 10.7705/biomedica.7676

**Published:** 2024-08-29

**Authors:** Carlos Álvarez-Dardet, Patricia Pérez-Wilson, Jorge Marcos-Marcos

**Affiliations:** 1 Grupo de Investigación en Salud Pública, Universidad de Alicante, Alicante, España Universidad de Alicante Grupo de Investigación en Salud Pública Universidad de Alicante Alicante Spain; 2 Programa de Salud y Medicina Familiar, Facultad de Medicina, Universidad de Concepción, Concepción, Chile Universidad de Concepción Programa de Salud y Medicina Familiar Facultad de Medicina Universidad de Concepción Concepción Chile; 3 Departamento de Psicología de la Salud, Universidad de Alicante, Alicante, España Universidad de Alicante Departamento de Psicología de la Salud Universidad de Alicante Alicante Spain

La relación entre salud y lo que ahora llamamos factores determinantes sociales” ha sido objeto de estudio científico desde hace tiempo. Los pioneros más conocidos en esta tarea fueron Friedrich Engels y Rudolf Virchow, quienes tuvieron una dedicación política importante y son conocidos, sobre todo, por razones ajenas a las que nos llevan a citarlos aquí: Engels, por su relación de amistad, intelectual y política con Karl Marx; Virchow, por ser considerado el padre de la patología celular, tan importante en biomedicina, y también por su fundación de la medicina social, siendo un gran defensor de la idea de que la salud de una población está intrínsecamente ligada a sus condiciones sociales y económicas y, por ende, de la medicina como ciencia social y de la política como medicina a escala poblacional ^1^^,^^2^.

Ya en los años 80 del siglo pasado, el informe Black sobre las desigualdades en salud en el Reino Unido incrementó la visibilidad política y el atractivo académico de la investigación en las desigualdades ^3^. Durante los años 80 y 90, se publicaron “informes Black” en muchos países y, sobre todo, la Organización Mundial de la Salud inició la tarea de potenciar la investigación sobre las desigualdades en salud. Para ello, se creó la Comisión de Determinantes Sociales de la Salud -constituida oficialmente en marzo de 2005, a partir de las decisiones de la 57^a^ Asamblea Mundial de la Salud- con la misión de reunir datos y evidencias sobre las causas sociales y ambientales de dichas desigualdades, y sobre posibles formas para corregirlas ^4^. Sin embargo, a pesar de que las desigualdades en salud se aceptan como problema prioritario, ningún país ha conseguido una disminución sensible de su importancia. Desgraciadamente, no nos hemos movido más allá de la retórica política.

En la actualidad, existe evidencia científica de calidad sobre la importancia causal que tienen las desigualdades sociales en la salud y el bienestar de las poblaciones, incluso, al aplicar a los hallazgos los criterios de causalidad del epidemiólogo inglés Austin Bradford Hill ^5^.

Sin embargo, en la mayoría de los países, las desigualdades en salud no han disminuido en la medida en la que se ha ido produciendo conocimiento científico relacionado con el tema. Tampoco lo han hecho las desigualdades sociales. En Europa, hemos confiado en que el aparato redistribuidor de la renta y los servicios públicos de salud de nuestros estados del bienestar conformarían una mano invisible que iría solucionando el problema, pero no ha sido así. En la literatura especializada, se acepta que para conseguir mayores cotas de igualdad en nuestras sociedades tenemos que desarrollar, no sólo políticas de redistribución, sino también, políticas de reconocimiento ^6^. Necesitamos mucha investigación sobre cómo poner en marcha programas que realmente consigan disminuir la importancia de las desigualdades.

En este sentido, en los últimos treinta años, al análisis crítico clásico de la clase social se han sumado dos ejes adicionales con gran valor explicativo en los estudios empíricos: el género, una noción que proviene del movimiento feminista, y la etnia, desarrollado a partir de la teoría crítica de la raza (dominación racial). Estos tres elementos (clase, género y raza/etnia) conforman parte del concepto de "interseccionalidad" (*intersectionality*), que permite comprender cómo se superponen las opresiones y vulnerabilidades a través de relaciones complejas que estructuran tanto el tejido social como las experiencias personales en la vida cotidiana ^7^.

Esto implica la conformación de un caleidoscopio de causas en la pérdida de salud, donde estas interaccionan entre sí de manera compleja. Para entender este fenómeno y, especialmente, para desarrollar intervenciones que puedan realmente solucionar estos problemas, necesitamos enfoques complementarios. La biomedicina, por sí sola, no está equipada con los instrumentos adecuados para reconocer y abordar esta realidad compleja y cambiante, ni para mejorar de manera efectiva la situación. Uno de los límites a los que nos enfrentamos es a la poca importancia que le da a los estudios cualitativos y a la investigación-acción.

Necesitamos redefinir urgentemente la teoría para luego poder transformar la práctica. Es crucial que abramos nuestras perspectivas y nos permeemos mediante la colaboración con otros campos, para comprender mejor la complejidad de las variables que influyen en la construcción y en el fortalecimiento de la salud. La colaboración más evidente es con las disciplinas de las ciencias sociales como la antropología, la sociología y la psicología, pero también, es vital integrar otras como la politología y la economía. Esto cobra aún más relevancia en los últimos años, ya que se considera que, entre los determinantes sociales de la salud, existen determinantes comerciales: aquellos relacionados con el daño en la salud producido por las políticas corporativas de la industria. Además, se ha aceptado la existencia de determinantes políticos, una noción que nuestro grupo definió hace años como “epidemiología política”, refiriéndose a “la influencia en la salud de las decisiones (o ausencia de decisiones) tomadas por instituciones derivadas del poder político” ^8^. -

En el área de salud pública, se observan algunos síntomas de revitalización en ese sentido, con un creciente trabajo teórico y empírico orientado a abordar estos determinantes desde un enfoque que permita identificar y movilizar los factores que generan salud y bienestar en las personas y en las comunidades. Teorías como la salutogénesis (sic.) y el modelo de activos para la salud, pueden ser propuestas valiosas al incorporar nuevas preguntas y formas de intervención que rescatan y ponen en valor los saberes y fortalezas de las personas y comunidades ^9^. En un mundo sanitario donde el modelo biomédico y su enfoque patogénico son hegemónicos, lo cual erosiona y desvaloriza estos saberes, el avanzar en esta dirección es más necesario que nunca.
